# Prevalence and morphological subtype distributions of anaemia in a Chinese rural population: the Henan Rural Cohort study

**DOI:** 10.1017/S1368980023000319

**Published:** 2023-06

**Authors:** Yiquan Zheng, Xiaotian Liu, Yaling He, Yinghao Yuchi, Hongfei Zhao, Linlin Li, Wenqian Huo, Zhenxing Mao, Jian Hou, Chongjian Wang

**Affiliations:** 1 Department of Epidemiology and Biostatistics, College of Public Health, Zhengzhou University, 100 Kexue Avenue, Zhengzhou, Henan 450001, People’s Republic of China; 2 Department of Occupational and Environmental Health Sciences, College of Public Health, Zhengzhou University, Zhengzhou, Henan, People’s Republic of China

**Keywords:** Epidemiology, Anaemia, Morphological subtypes, Prevalence, Rural population

## Abstract

**Objective:**

This study aimed to evaluate the recent prevalence and the distributions of morphological subtypes of anaemia in the rural population.

**Design:**

Anaemia was defined according to the WHO and the Chinese criteria, and the morphological subtypes of anaemia were classified based on the erythrocyte parameters. The age-standardised prevalence was calculated according to the data of the Population Census 2010 in China.

**Setting:**

A cross-sectional study in Henan Province.

**Participants:**

33 585 subjects aged 18–79 years old.

**Results:**

The standardised prevalence of anaemia across the WHO and the Chinese definitions was 13·63 % and 5·45 %, respectively. Regardless of which criteria was used, the standardised prevalence of anaemia was higher among women than among men and that increased with age in men, while markedly decreased after menopause in women. There were shifts in morphological patterns of anaemia using the WHO and the Chinese criteria that the standardised prevalence of microcytic anaemia was 3·74 % and 2·97 %, normocytic anaemia was 9·20 % and 2·34 %, and macrocytic anaemia was 0·75 % and 0·14 %, respectively. Besides, there were differences in the influencing factors of anaemia according to different criteria or gender. However, age, education level and renal damage were consistently significantly associated with anaemia in all participants.

**Conclusions:**

Anaemia may still be a serious health problem in rural China. It is necessary to reformulate prevention and management strategies to reduce the disease burden of anaemia.

Anaemia is a syndrome that occurs when an insufficiency in the number of healthy erythrocytes results in inadequate oxygen supply to vital tissues^(1)^, and anaemia is diagnosed when the Hb concentration is under a specific threshold^(2)^. Cumulative evidence has shown that anaemia could increase the risk of CVD outcomes^(3)^, impair cognitive function^(4)^ and reduce the healthy quality of life^(5)^. It was estimated that 1·8 billion people (23·2 % of the world’s population) were anaemic in 2019. Although the prevalence of anaemia decreased by 13·40 % from 1990 to 2019, anaemia was still a major health problem, especially in developing countries^(6)^. Besides, a previous Chinese national study covering thirty-one provinces indicated that the prevalence of anaemia among Chinese rural people dropped from 22·2 % in 2002 to 9·7 % in 2012^(7)^. However, there has been a paucity of large-scale investigations of Chinese rural people in recent years. Furthermore, with the economy’s development and the changing lifestyle, the potential influencing factors of anaemia should be explored.

The distributions of Hb concentrations among different ethnic groups are different^(8)^. Besides, the current criteria recommended by the WHO was from five studies of the predominantly White population that lacked data from other countries and races^(9)^, so the WHO cut-off may not be suitable for the Chinese population. Meanwhile, the data from various populations and low-income and middle-income countries are urgently needed to re-examine existing Hb thresholds to define anaemia^(10)^. Therefore, the current study compared the prevalence of anaemia between the Chinese and the WHO criteria to inform public health programmes.

In addition, although several prior studies have investigated the prevalence of anaemia in various countries^(7,11,12)^, the evidence on the prevalence of morphological subtypes of anaemia is lack. The classification of anaemia according to morphology can provide clues for differential diagnosis to help find the aetiology quickly^(13,14)^. Information on the prevalence of morphological subtypes of anaemia in different criteria and population subgroups is needed to formulate relevant health policy^(11)^. The corresponding strategies can be formulated according to the prevalence variation patterns of different populations. Meanwhile, the diagnostic threshold can be adjusted based on the shifts in the prevalence of anaemia using different criteria.

Therefore, the purposes of the current study were to estimate the prevalence of anaemia with different criteria and different morphological anaemia subtypes classification methods and to explore the potential related factors in the Chinese rural population.

## Methods

### Study population

The study population was from the Henan Rural Cohort study, and the detailed information has been described elsewhere^(15)^. From July 2015 to September 2017, a total of 39 259 participants aged 18–79 years from five counties of Henan Province in China were recruited with a multistage, stratified cluster sampling method. After the exclusion of subjects with missing Hb concentration data (*n* 5287), having severe diseases (kidney failure or malignant tumour) (*n* 326) and pregnant women (*n* 61), a total of 33 585 participants were included in the final analyses. The protocol of this prospective cohort study was approved by the Zhengzhou University Life Science Ethics Committee, and written informed consent from each participant was obtained.

### Assessment of covariates

All participants completed a structured questionnaire including demographic characteristics, behavioural lifestyles and medical history. The demographic characteristics included age, sex, education level (primary school or below, junior school, and high school or above), marital status (married/cohabiting and widowed/single/divorced/separated), and per capita monthly income was classified into three categories: <500 RMB (renminbi, the Chinese currency and the average exchange rate for USD/RMB from 2015 to 2017 is 6·54), 500–1000 RMB and ≥1000 RMB. Behavioural lifestyles included smoking and drinking status, physical activity, high-fat diet, and adequate vegetable and fruit intake. According to the smoking index^(16)^ and the daily alcohol intake guidelines^(17,18)^, smoking and drinking status was grouped into never, light, moderate and heavy. Physical activity was classified into low, moderate and high levels based on the International Physical Activity Questionnaire^(19)^. Besides, following the Chinese dietary guidelines, high-fat diet and adequate vegetable and fruit intake were defined as the average daily intake of livestock and/or poultry meat ≥ 75 g^(18)^ as well as vegetable and fruit ≥ 500 g^(20)^, respectively. Furthermore, blood samples, blood pressure measurements and anthropometric data were also collected. Hypertension was defined as systolic blood pressure ≥ 140 mmHg and/or diastolic blood pressure ≥ 90 mmHg, or self-reported hypertension or using antihypertension medication during the last 2 weeks^(21)^. BMI was classified into four groups: underweight (BMI < 18·5 kg/m^2^), normal weight (18·5 ≤ BMI < 24 kg/m^2^), overweight (24·0 kg/m^2^ ≤ BMI < 28·0 kg/m^2^) and obesity (BMI ≥ 28·0 kg/m^2^)^(22)^. After at least 8 h of overnight fasting, venous blood samples were collected to obtain haematological data. The definition of type 2 diabetes mellitus (T2DM) was the fasting blood glucose ≥ 7·0 mmol/l, or having a self-reported history of diabetes or using insulin or antidiabetic medication during the last 2 weeks^(23)^. The definition of dyslipidemia was the serum total cholesterol ≥ 6·22 mmol/l or TAG ≥ 2·26 mmol/l or HDL-cholesterol < 1·04 mmol/l or LDL-cholesterol ≥ 4·14 mmol/l or self-reported dyslipidemia or using lipid-lowering drugs during the last 2 weeks^(24)^. The Chronic Kidney Disease Epidemiology Collaboration equation was used to calculate the estimated glomerular filtration rate (eGFR)^(25)^, and eGFR < 60 ml/min/1·73 m^2^ was defined as renal damage^(26)^.

### Definition of anaemia and classification into morphological subtypes

Haematological data were estimated by Sysmex XS-500i automatic biochemical analyser. The measured Hb concentrations were adjusted depending on the specific smoking status to correct the effect of smoking on Hb^(8)^. The specific adjustment values were based on the detailed categorisation of smoking status (< 20 cigarettes smoked per d or unknown amount, -3 g/l; ≥20 and <40 cigarettes smoked per d, -5 g/l; ≥ 40 cigarettes smoked per d, -7 g/l). In the current study, anaemia was defined according to the WHO and the Chinese criteria: Hb concentrations lower than 130/120 g/l (for men/women)^(8)^ and 120/110 g/l, respectively. After the diagnosis of anaemia based on the different cut-off values, anaemia was subclassified based on mean corpuscular volume (MCV). Microcytic anaemia was determined as MCV < 80 fL, normocytic anaemia as MCV between 80 and 100 fL, and macrocytic anaemia as MCV > 100 fL^(27)^. The morphological subtypes were further classified according to the methods proposed by Bessman^(28)^ and Wintrobe^(29)^, respectively. Briefly, the methods used additional erythrocyte parameters based on MCV classification. Bessman used red blood cell volume distribution width CV (RDW-CV)^(28)^, and RDW-CV ≥ 14·5 % was considered anisocytosis, while the method proposed by Wintrobe used mean corpuscular haemoglobin and mean corpuscular haemoglobin concentration. The details of these two classifications were shown in Supplemental Tables 1 and 2. In the current study, two anaemia definitions and three morphological anaemia subtypes classification methods were used to observe whether the variations in anaemia prevalence change with different definitions and categories.

### Statistical analyses

Continuous variables were described as mean ± sd, and the differences between groups were compared with Student’s *t* test. Categorical variables were expressed as frequency (percentage), and the differences were assessed with Pearson’s Chi-square test. The age-standardised prevalence of anaemia was calculated according to the data of the Population Census 2010 in China. The logistic regression model was used to estimate the association between characteristics and anaemia (adjusted OR (aOR) and 95 % CI). Based on previous studies^(30,31)^, biological relevance and findings of the univariate analyses, several variables were adjusted in the multivariable adjustment models: age, education level, marital status, per capita monthly income, smoking status, drinking status, physical activity, high-fat diet, adequate vegetable and fruit intake, BMI group, hypertension, T2DM, dyslipidemia, and renal damage. Stratified analysis was conducted on women on the basis of their self-reported menopausal status. All statistical analyses were performed using SPSS 22.0, and two-sided *P*-values <0·05 were considered statistically significant.

## Results

### Characteristic of participants

Table 1 and Supplemental Table 3 present the characteristics of 33 585 participants by gender using the WHO and the Chinese definitions of anaemia, respectively. In brief, participants with low education level, widowed/single/divorced/separated and lower income level accounted for a higher proportion of men with anaemia in the WHO and the Chinese criteria. Moreover, anaemic men tend to be older, and heavy smoking, never drinking, lower level of eGFR and BMI, without high-fat diet. However, the difference was that anaemic men had adequate vegetable and fruit intake and were without hypertension or dyslipidemia according to the WHO anaemia definition. Besides, anaemic men had low physical activity and never smoking based on the Chinese anaemia definition. For women, anaemic women tend to be younger, have lower BMI, higher eGFR levels and a higher proportion of married/cohabiting, high-fat diet, as well as a lower proportion of hypertension, T2DM, and dyslipidemia. In addition, differences in physical activity and adequate vegetable and fruit intake were observed according to the WHO criteria but not the Chinese criteria.

**Table 1:** Characteristics of study participants by gender according to the WHO anaemia definition

Variables	Men (*n* 13 574)					Women (*n* 20 011)				
	Anaemia		Non-anaemia		*P*-value	Anaemia		Non-anaemia		*P*-value
	Mean	sd	Mean	sd		Mean	sd	Mean	sd	
Age (years)	63·15	9·68	55·76	12·40	<0·001	51·22	12·4	55·30	12·02	<0·001
	*n*	%	*n*	%		*n*	%	*n*	%	
Education level					<0·001					<0·001
Primary school or below	447	50·85	4030	31·74		1560	47·52	8645	51·68	
Junior school	346	39·36	5909	46·55		1288	39·23	5878	35·14	
High school or above	86	9·79	2756	21·71		435	13·25	2205	13·18	
Marital status					<0·001					0·007
Married/cohabiting	744	84·64	11 522	90·76		2999	91·35	15 022	89·80	
Widowed/single/divorced/separated	135	15·36	1173	9·24		284	8·65	1706	10·20	
Per capita monthly income					<0·001					0·046
<500 RMB	420	47·78	4454	35·09		1125	34·27	5884	35·17	
500–1000 RMB	258	29·35	4017	31·64		1057	32·19	5600	33·48	
≥1000 RMB	201	22·87	4224	33·27		1101	33·54	5244	31·35	
Smoking status					<0·001					0·823
Never	233	26·51	4068	32·04		3273	99·7	16 661	99·6	
Light	91	10·35	1832	14·43		6	0·18	39	0·23	
Moderate	88	10·01	1469	11·57		1	0·03	11	0·07	
Heavy	467	53·13	5326	41·95		3	0·09	17	0·10	
Drinking status					<0·001					0·553
Never	500	56·88	5974	47·06		3210	97·78	16 324	97·58	
Light	229	26·05	4012	31·60		62	1·89	317	1·90	
Moderate	71	8·08	1487	11·71		8	0·24	57	0·34	
Heavy	79	8·99	1222	9·63		3	0·09	30	0·18	
Physical activity					0·753					0·044
Low	305	34·70	4440	34·97		933	28·41	5043	30·15	
Moderate	258	29·35	3581	28·21		1512	46·06	7325	43·79	
High	316	35·95	4674	36·82		838	25·53	4360	26·06	
High-fat diet					<0·001					0·006
No	708	80·55	9542	75·16		2746	83·64	14 303	85·50	
Yes	171	19·45	3153	24·84		537	16·36	2425	14·50	
Adequate vegetable and fruit intake					<0·001					<0·001
No	427	48·58	6986	55·04		1681	51·20	9410	56·25	
Yes	452	51·42	5707	44·96		1602	48·80	7318	43·75	
Hypertension	236	26·85	4168	32·83	<0·001	639	19·46	5736	34·29	<0·001
T2DM	76	8·65	1106	8·71	0·947	180	5·48	1658	9·91	<0·001
Dyslipidemia	248	28·21	5274	41·54	<0·001	898	27·35	6417	38·36	<0·001
	Mean	sd	Mean	sd		Mean	sd	Mean	sd	
BMI (kg/m^2^)	22·55	3·40	24·68	3·44	<0·001	24·20	3·55	25·10	3·60	<0·001
eGFR (ml/min/1·73 m^2^)	93·14	14·8	99·27	12·89	<0·001	103·35	15·54	100·23	13·18	<0·001
Hb (g/l)	121·62	9·93	150·29	11·98	<0·001	107·97	12·06	134·64	11·59	<0·001

RMB, renminbi, the Chinese currency, and the average exchange rate for USD/RMB from 2015 to 2017 is 6.54; T2DM, type 2 diabetes mellitus eGFR, estimated glomerular filtration rate.

### The standardised prevalence of anaemia

**Fig. 1:**
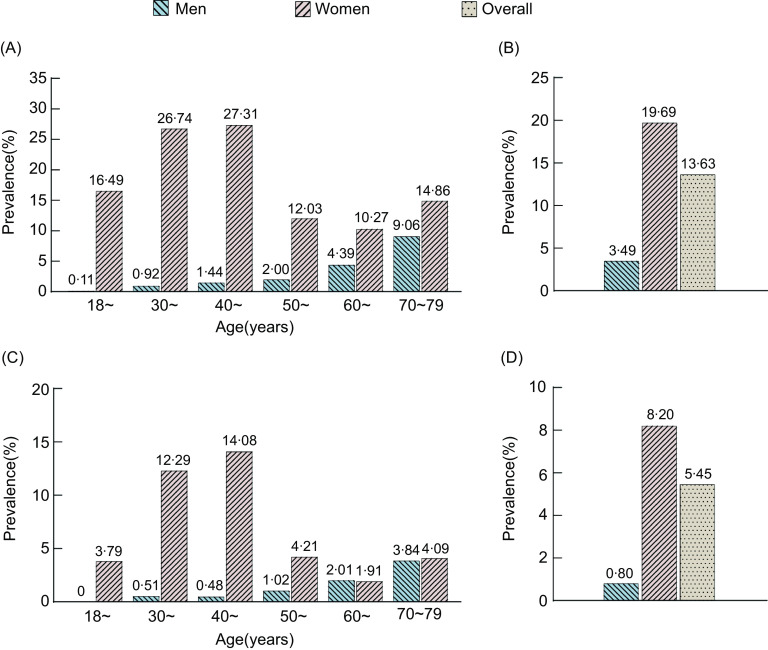
Age-specific prevalence of anaemia by the WHO definition and the Chinese definition among Chinese rural population. (The age-standardised prevalence of anaemia by the WHO definition (Panel A) and the Chinese definition (Panel C) among men and women was shown, according to age. The age-standardised prevalence of anaemia by the WHO definition and the Chinese definition was shown in Panels B and D, respectively)

The overall standardised prevalence of anaemia was 13·63 % and 5·45 % to the WHO and the Chinese criteria, respectively. The age-standardised prevalence of anaemia was significantly higher among women than men in both diagnostic definitions of anaemia. The findings observed that the age-standardised prevalence increased with age among men and only women aged ≥ 60 years. Meanwhile, that was substantially higher in women of reproductive age (18–49 years) than in other age groups (Fig. 1). Besides, the prevalence of anaemia according to other characteristics was described in Table 2 and Supplemental Table 4.

**Table 2:** The prevalence and 95 % CI for anaemia among characteristics according to the WHO definition

Variables	Men (*n* 13 574)				Women (*n* 20 011)			
	*n*	Prevalence	95 % CI	*P*-value	*n*	Prevalence	95 % CI	*P*-value
Education level				<0·001				<0·001
Primary school or below	4477	9·98	9·11, 10·86		10 205	15·29	14·59, 15·98	
Junior school	6255	5·53	4·96, 6·10		7166	17·97	17·08, 18·86	
High school or above	2842	3·03	2·40, 3·66		2640	16·48	15·06, 17·89	
Marital status				<0·001				0·007
Married/cohabiting	12 266	6·07	5·64, 6·49		18 021	16·64	16·10, 17·19	
Widowed/single/divorced/separated	1308	10·32	8·67, 11·97		1990	14·27	12·73, 15·81	
Per capita monthly income				<0·001				0·046
<500 RMB	4874	8·62	7·83, 9·41		7009	16·05	15·19, 16·91	
500–1000 RMB	4275	6·04	5·32, 6·75		6657	15·88	15·00, 16·76	
≥1000 RMB	4425	4·54	3·93, 5·16		6345	17·35	16·42, 18·28	
Smoking status				<0·001				0·823
Never	4301	5·42	4·74, 6·09		19 934	16·42	15·90, 16·93	
Light	1923	4·73	3·78, 5·68		45	13·33	3·01, 23·66	
Moderate	1557	5·65	4·50, 6·80		12	8·33	-10·01, 26·67	
Heavy	5793	8·06	7·36, 8·76		20	15·00	-2·15, 32·15	
Drinking status				<0·001				0·553
Never	6474	7·72	7·07, 8·37		19 534	16·43	15·91, 16·95	
Light	4241	5·40	4·72, 6·08		379	16·36	12·62, 20·10	
Moderate	1558	4·56	3·52, 5·59		65	12·31	4·10, 20·51	
Heavy	1301	6·07	4·77, 7·37		33	9·09	-1·26, 19·44	
Physical activity				0·753				0·044
Low	4745	6·43	5·73, 7·13		5976	15·61	14·69, 16·53	
Moderate	3839	6·72	5·93, 7·51		8837	17·11	16·32, 17·90	
High	4990	6·33	5·66, 7·01		5198	16·12	15·12, 17·12	
High-fat diet				<0·001				0·006
No	10 250	6·91	6·42, 7·40		17 049	16·11	15·55, 16·66	
Yes	3324	5·14	4·39, 5·90		2962	18·13	16·74, 19·52	
Adequate vegetable and fruit intake				<0·001				<0·001
No	7413	5·76	5·23, 6·29		11 091	15·16	14·49, 15·82	
Yes	6159	7·34	6·69, 7·99		8920	17·96	17·16, 18·76	
BMI group				<0·001				0·001
Underweight	370	22·16	17·91, 26·41		463	26·57	22·53, 30·60	
Normal	5851	9·2	8·45, 9·94		7810	19·77	18·89, 20·65	
Overweight	5171	3·58	3·07, 4·08		7999	14·76	13·99, 15·54	
Obesity	2141	3·13	2·39, 3·87		3687	11·47	10·44, 12·50	
Hypertension	4404	5·36	4·69, 6·02	<0·001	6375	10·02	9·29, 10·76	<0·001
T2DM	1182	6·43	5·03, 7·83	0·947	1838	9·79	8·43, 11·15	<0·001
Dyslipidemia	5522	4·49	3·94, 5·04	<0·001	7315	12·28	11·52, 13·03	<0·001
Renal damage	120	29·17	20·92, 37·42	<0·001	160	35·63	28·12, 43·13	<0·001

RMB, renminbi, the Chinese currency, and the average exchange rate for USD/RMB from 2015 to 2017 is 6.54; T2DM, type 2 diabetes mellitus.

### The standardised prevalence of morphological subtypes

**Fig. 2:**
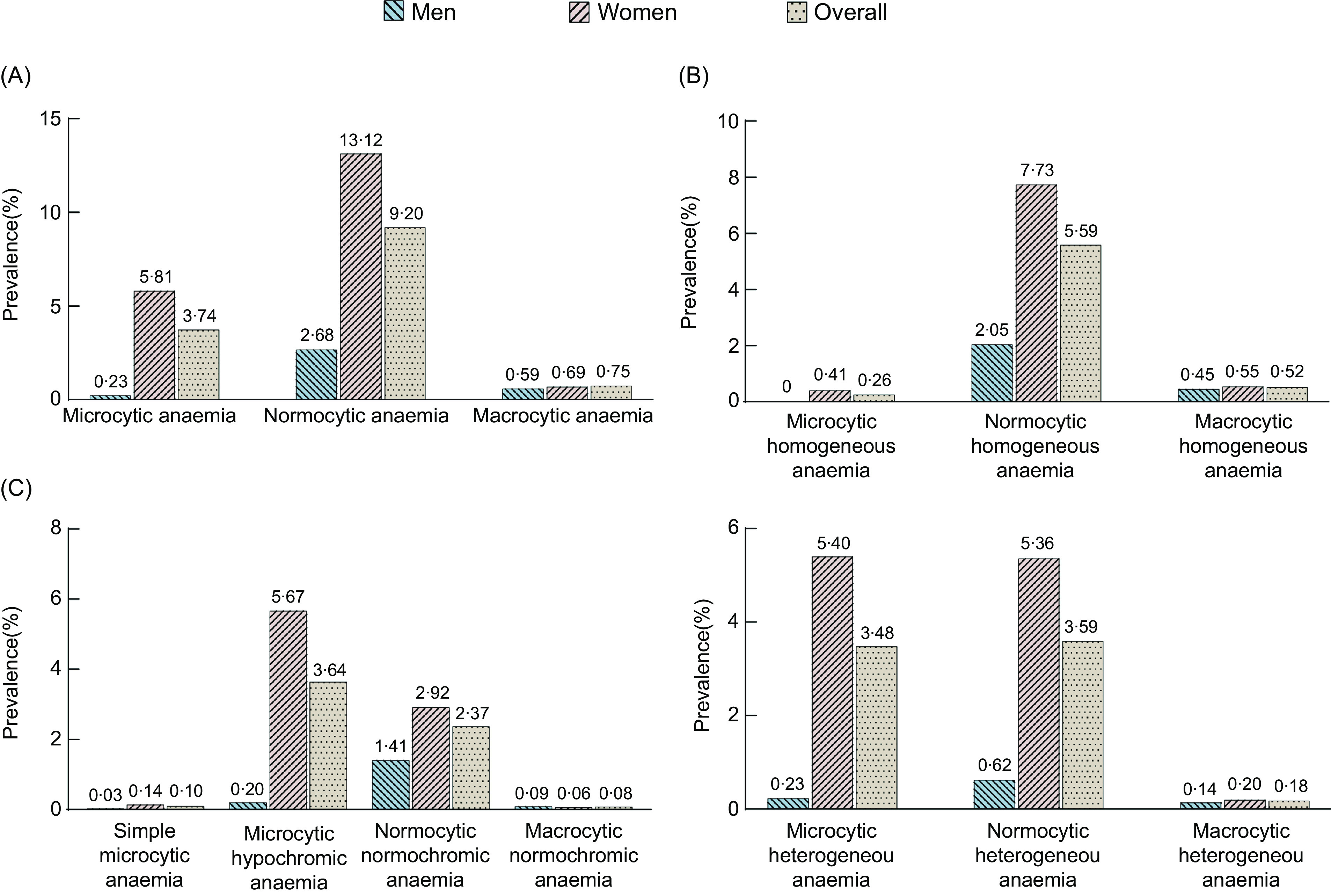
The age-standardised prevalence of morphological subtypes of anaemia according to the WHO definition. (The age-standardised prevalence of morphological subtypes of anaemia classified based on mean corpuscular volume was shown in Panel A. Panels B and C showed the age-standardised prevalence of morphological subtypes of anaemia according to the methods proposed by Bessman and Wintrobe, respectively)

Figure 2 and Supplemental Fig 1 show the prevalence distributions of morphological subtypes of anaemia across the WHO and the Chinese definitions, respectively. The standardised prevalence of microcytic, normocytic and macrocytic anaemia in total participants was 3·74 %, 9·20 % and 0·75 % by the WHO definition (Fig. 2(a)), while that was 2·97 %, 2·34 % and 0·14 % by the Chinese criteria, respectively (see online Supplemental Fig. 1(a)). The age-standardised prevalence of microcytic and normocytic anaemia was significantly higher among women than men. However, the standardised prevalence of macrocytic anaemia was slightly higher among women than among men according to the WHO criteria and even had the same standardised prevalence according to the Chinese criteria. Furthermore, both in the WHO and the Chinese definitions of anaemia, the age-standardised prevalence of microcytic anaemia and normocytic anaemia was highest among women aged 30–49 years, while macrocytic anaemia prevalence was highest among women aged 70–79 years. However, the age-standardised prevalence of these three subtypes of anaemia was observed to increase with age among men (see online Supplemental Figs. 2(a) and 3(a)).

Bessman’s classification method further classified each microcytic, normocytic and macrocytic anaemia subtype into homogeneous or heterogeneous categories. As defined by the WHO or the Chinese criteria, the standardised prevalence of microcytic heterogeneous anaemia, microcytic homogeneous anaemia, normocytic heterogeneous anaemia and normocytic homogeneous anaemia was higher among women than among men (Fig. 2(b) & see online Supplemental Fig. 1(b)), which was highest among women aged 30–49 years but among men aged 70–79 years (see online Supplemental Figs. 2(b) and 3(b)). Moreover, the standardised prevalence of macrocytic heterogeneous anaemia and macrocytic homogeneous anaemia was also higher among women than among men, except for the prevalence of macrocytic homogeneous anaemia by the Chinese definitions. However, among men and women, the highest prevalence of macrocytic heterogeneous anaemia and macrocytic homogeneous anaemia was observed in the 70–79 age group.

The method proposed by Wintrobe additionally used mean corpuscular haemoglobin and mean corpuscular haemoglobin concentration to further classify anaemia as simple microcytic anaemia, microcytic hypochromic anaemia, normocytic normochromic anaemia and macrocytic normochromic anaemia. According to the WHO and the Chinese definitions, the standardised prevalence of the first three subtypes of anaemia was higher among women than among men, but that was the opposite in the macrocytic normochromic anaemia (Fig. 2(c) & see online Supplemental Fig. 1(c)). Besides, the highest prevalence of microcytic hypochromic anaemia and normocytic normochromic anaemia was in the 70–79 age group among men and 30–49 age group among women, respectively (see online Supplemental Figs. 2(c) and 3(c)). Furthermore, simple microcytic anaemia had the highest prevalence in the 60–69 age group among men and the 40–49 age group among women, while macrocytic normochromic anaemia in the 70–79 age group among men and women.

### Related factors for anaemia

Across the WHO definition, adequate vegetable and fruit intake, being underweight, and renal damage were all significantly positively associated with anaemia prevalence among men and women. In contrast, higher educational level, overweight and obesity, hypertension, and dyslipidemia were significantly negatively linked to anaemia prevalence among both men and women. Additionally, men who were elder and had heavy smoking were related to a higher prevalence of anaemia, while men with higher income levels were related to a lower prevalence of anaemia. Meanwhile, women who were younger or without T2DM were more likely to suffer from anaemia (Table 3).

**Table 3:** Gender-specific multivariable logistic regression analysis for the influencing factors for anaemia according to the WHO definition

Variables	Men			Women		
	aOR	95 % CI	*P*-value	aOR	95 % CI	*P*-value
Age	1·043	1·035, 1·051	<0·001	0·974	0·970, 0·978	<0·001
Education level						
Primary school or below	Ref	1·000		Ref	1·000	
Junior school	0·812	0·692, 0·952	0·010	0·858	0·783, 0·940	0·001
High school or above	0·610	0·473, 0·786	<0·001	0·600	0·523, 0·689	<0·001
Marital status						
Married/cohabiting	Ref	1·000		Ref	1·000	
Widowed/single/divorced/separated	1·159	0·941, 1·427	0·165	0·999	0·869, 1·148	0·985
Per capita monthly income						
<500 RMB	Ref	1·000		Ref	1·000	
500–1000 RMB	0·916	0·774, 1·084	0·308	0·892	0·811, 0·981	0·018
≥1000 RMB	0·812	0·675, 0·977	0·027	0·936	0·850, 1·031	0·182
Smoking status						
Never	Ref	1·000		Ref	1·000	
Light	1·081	0·833, 1·403	0·560	0·945	0·388, 2·300	0·900
Moderate	1·204	0·924, 1·570	0·169	0·479	0·061, 3·759	0·484
Heavy	1·383	1·160, 1·649	<0·001	1·335	0·382, 4·664	0·651
Drinking status						
Never	Ref	1·000		Ref	1·000	
Light	0·888	0·747, 1·056	0·180	0·823	0·622, 1·089	0·172
Moderate	0·769	0·589, 1·005	0·055	0·637	0·300, 1·352	0·241
Heavy	0·946	0·728, 1·228	0·675	0·510	0·151, 1·720	0·278
High-fat diet	0·997	0·829, 1·199	0·976	0·987	0·887, 1·098	0·808
Adequate vegetable and fruit intake	1·265	1·095, 1·462	0·001	1·143	1·057, 1·236	0·001
Physical activity						
Low	Ref	1·000		Ref	1·000	
Moderate	1·042	0·870, 1·249	0·653	0·999	0·910, 1·096	0·982
High	1·022	0·860, 1·215	0·804	0·977	0·879, 1·085	0·661
BMI group						
Normal	Ref	1·000		Ref	1·000	
Underweight	2·227	1·694, 2·927	<0·001	1·414	1·136, 1·760	0·002
Overweight	0·450	0·376, 0·539	<0·001	0·786	0·721, 0·857	<0·001
Obesity	0·470	0·356, 0·622	<0·001	0·642	0·569, 0·725	<0·001
Hypertension	0·763	0·645, 0·904	0·002	0·628	0·567, 0·694	<0·001
T2DM	1·264	0·979, 1·632	0·072	0·749	0·635, 0·883	0·001
Dyslipidemia	0·837	0·709, 0·987	0·034	0·769	0·704, 0·839	<0·001
Renal damage	4·535	2·927, 7·025	<0·001	5·168	3·683, 7·252	<0·001

aOR, adjusted OR; RMB, renminbi, the Chinese currency, and the average exchange rate for USD/RMB from 2015 to 2017 is 6·54; T2DM, type 2 diabetes mellitus.

According to the Chinese anaemia criteria, multivariable logistic regression analyses showed that older, widowed/single/divorced/separated, underweight, T2DM and renal damage were significantly associated with higher risks of anaemia, while higher education level, light smoking and overweight but not obesity were associated with lower risks of anaemia in men. Additionally, older, with higher education and income level, widowed/single/divorced/separated, hypertension, diabetes, and dyslipidemia were associated with lower risks of anaemia, whereas renal damage was significantly associated with an elevated risk of anaemia in women (see online Supplemental Table 5).

After stratified by self-reported menopausal status in women, we found age became positively associated with anaemia, but the association could not be observed among post-menopausal women according to Chinese anaemia criteria. Besides, the stratified analysis also showed that the included influencing factors mainly affected post-menopausal women (see online Supplemental Tables 6 and 7).

## Discussion

The present study evaluated the recent prevalence of anaemia and the distributions of morphological subtypes based on the WHO and the Chinese definitions of anaemia, and adding a new piece of evidence to the current burden of anaemia among the rural Chinese population. The standardised prevalence among total participants of anaemia across the WHO and the Chinese definitions were 13·63 % and 5·45 %, respectively. Furthermore, the logistic regression analyses found different related factors associated with anaemia in men and women.

There are plenty of aetiologies of anaemia, and the morphological classification of anaemia can provide clues for diagnosing different aetiology, which can narrow the range of differential diagnoses and reduce healthcare costs^(13)^. For example, microcytic anaemia is most commonly caused by Fe deficiency^(14)^, while chronic kidney disease is characterised by normocytic normochromic anaemia^(32)^. This study observed the distribution of the aetiologies of anaemia by investigating the distribution of the morphological classification of anaemia. Besides, the distribution of these subtypes may also provide information to develop targeted strategies to reduce the burden of anaemia.

Recognising the treatable causes of anaemia is critical to reducing the burden. Several treatable causes include nutrient deficiency, renal damage and hemolysis in normocytic anaemia. Meanwhile, although Fe and vitamin B_12_/folate deficiencies are usually linked to microcytic and macrocytic anaemia, they are characterised by normocytic anaemia in the early stages of nutrient deficiency^(14,28)^. However, the prevalence of normocytic anaemia was markedly lower in the Chinese criteria than in the WHO criteria, which may reduce the opportunity to find anaemia with treatable causes. Besides, a similar prevalence of microcytic anaemia was observed in both criteria. Microcytic anaemia may lead to a strong reduction of Hb. A previous study indicated that macrocytic anaemia was associated with higher mortality^(27)^, but the prevalence of macrocytic anaemia was pretty low in both criteria.

The classification methods proposed by Bessman and Wintrobe further divided the causes of anaemia by using additional erythrocyte parameters. Besides, there were shifts in morphological patterns due to fractional mild anaemia could not be detected in a more stringent Hb threshold (Chinese criteria). Combining these two methods and these shifts, the aetiologies which lead to this fraction of mild anaemia might be found. The information could be further provided to adjust public health policies by finding the treatable aetiologies from these undetected aetiologies^(14)^.

The shifts in morphological subtypes’ prevalence of anaemia between the two criteria showed that the prevalence of normocytic anaemia changed the most, but the change in the prevalence of normocytic heterogeneous anaemia was less than normocytic homogeneous anaemia and normocytic normochromic anaemia. This phenomenon might mean that acute blood loss, hemolysis and renal damage were less likely to be detected than early nutrient deficiency and sideroblastic anaemia^(28,32)^. Between the two anaemia criteria, the prevalence of all macrocytic subtypes of anaemia, simple microcytic anaemia and microcytic homogeneous anaemia also varied greatly. The causes of macrocytic anaemia including hemolysis, megaloblastic anaemia and aplastic anaemia; meanwhile, infection and poisoning could cause simple microcytic anaemia, while thalassemia and anaemia of chronic diseases were for microcytic homogeneous anaemia^(14,28,29)^. Additionally, the prevalence of microcytic hypochromic anaemia and microcytic heterogeneous anaemia had minimal changes while using different definitions, and the etiology includes Fe deficiency (both microcytic anaemia), thalassemia and Hemoglobin H disease^(28,29)^. Otherwise, Fe deficiency anaemia is most common in microcytic anaemia and has the highest disease burden among anaemia^(6,14)^.

Although the variation of anaemia prevalence between the two definitions marked, the age-specific and gender-specific prevalence of anaemia had a similar changing trend across the two criteria. The prevalence of most morphological subtypes of anaemia was also higher in women than men. A previous study conducted in thirteen Korean cities showed consistent results^(33)^. For women of reproductive age, the high anaemia prevalence may be attributed to their physiological vulnerability^(34)^, which results in a much higher prevalence among women in this age group than in other age groups, and this population is the focus of most current studies^(1,35)^. With the end of menstruation, this physical vulnerability ceases to exist^(36)^. However, high anaemia prevalence is also common in the elder due to deterioration of the physical condition and the occurrence of co-morbidity^(37,38)^. That explains why the prevalence of anaemia increased among men and post-menopausal women. The stratified analysis also showed that age was a risk factor for anaemia in men, pre-menopausal women and post-menopausal women.

Multivariate logistic regression analyses indicated the associations of demographic, lifestyle and chronic diseases with anaemia, and these findings were in line with the results of previous studies^(12,39–42)^. The mechanism proposed to explain the positive associations of adequate vegetable and fruit intake with anaemia was mainly attributed to inadequate intakes of some essential nutrients which were not easily obtained from plant sources^(12)^. Meanwhile, overweight and obese people may be in a state of nutritional surplus, where they are more likely to get adequate micronutrients^(43)^. Besides, previous studies indicated that obesity, hypertension and T2DM were associated with erythrocytosis^(44,45)^. Moreover, a study based on the general population showed that BMI, blood pressure, total cholesterol, LDL-cholesterol and TAG were higher in individuals with erythrocytosis significantly^(46)^. However, only T2DM was positively associated with anaemia in men based on the Chinese criteria. T2DM can cause systemic inflammation^(14)^ and renal damage^(47)^ and further induce anaemia. The inflammation can block the Fe cycle, shorten erythrocyte lifespan, and reduce erythropoietin production and biological activity^(48)^. Consistent with previous studies, renal disease was linked to anaemia by impairing the ability of erythropoietin production and further reducing erythrocyte production^(32,49)^. The main pathway was the reduction of the ability of erythropoietin production by inducing the erythropoietin-producing cells to differentiate into myofibroblasts^(50)^.

The findings of the current study have several health policy implications. Firstly, microcytic anaemia had the most stable prevalence, suggesting that the prevention and control of Fe deficiency anaemia (the most common microcytic anaemia) should continue. Secondly, a considerable number of participants suffered from normocytic anaemia with mild Hb reduction. Future efforts to reduce the burden of anaemia could focus on that. Finally, the prevalence of different anaemia subtypes had a similar variation pattern. Women of reproductive age should be considered first and older persons second in policy formulation, regardless of the subtype.

To the best of our knowledge, this was the first study that compared the prevalence of anaemia by the WHO and the Chinese definitions and evaluated the distributions of morphological subtypes of anaemia in the Chinese rural population. Furthermore, this study used a variety of definitions and classifications of anaemia, had a large sample size, and strict quality control and quality assurance. However, there were some limitations. As the present study was a cross-sectional design, associations of some related factors with anaemia may be reverse causality. Therefore, more prospective studies are needed to validate our results. Moreover, only diagnosing aetiologies by morphological subtypes was not definitive due to the unavailability of data such as ferritin (defining Fe deficiency anaemia) in the current study. However, these subtypes can provide information to narrow the range of aetiologies in the initial diagnosis and save medical resources^(14)^. Besides, the results from a portion of the Chinese population might not be representative of the entire rural region of China. However, to some extent, the results of the current study can reflect the prevalence of anaemia in the Chinese rural areas due to the large sample size as well as the Henan rural population accounts for 8·9 % of the Chinese rural population^(15)^. Due to the influence of dietary factors on anaemia, future studies should include the population in other regions for research. However, the results of this large sample study likely reflect the prevalence of anaemia in rural areas of China.

## Conclusion

In summary, the estimated standardised prevalence of anaemia was 13·63 % and 5·45 % in rural China across the WHO and the Chinese definitions, and the prevalence was higher among women than among men. The prevalence of microcytic anaemia was the most stable, while normocytic anaemia changed the greatest when different definitions were used. Several demographics, lifestyles and metabolic factors were associated with anaemia risk. Our results suggested that anaemia may still be a serious public health problem in the current population. Therefore, effective strategies are necessary to minimise the effect of the influencing factors for anaemia and decrease the prevalence of anaemia, especially in rural regions with high prevalence.

## Supplementary material

For supplementary material accompanying this paper visit https://doi.org/10.1017/S1368980023000319. click here to view supplementary material

## References

[1] Kinyoki D , Osgood-Zimmerman AE , Bhattacharjee NV et al. (2021) Anemia prevalence in women of reproductive age in low- and middle-income countries between 2000 and 2018. Nat Med 27, 1761–1782. 34642490 10.1038/s41591-021-01498-0 PMC8516651

[2] Sachdev HS , Porwal A , Acharya R et al. (2021) Haemoglobin thresholds to define anaemia in a national sample of healthy children and adolescents aged 1–19 years in India: a population-based study. Lancet Glob Health 9, e822–e831. 33872581 10.1016/S2214-109X(21)00077-2 PMC7612991

[3] Sarnak MJ , Tighiouart H , Manjunath G et al. (2002) Anemia as a risk factor for cardiovascular disease in the atherosclerosis risk in communities (ARIC) study. J Am Coll Cardiol 40, 27–33. 12103252 10.1016/s0735-1097(02)01938-1

[4] Wolters FJ , Zonneveld HI , Licher S et al. (2019) Hemoglobin and anemia in relation to dementia risk and accompanying changes on brain MRI. Neurology 93, e917–e926. 31366722 10.1212/WNL.0000000000008003 PMC6745727

[5] Wouters H , van der Klauw MM , de Witte T et al. (2019) Association of anemia with health-related quality of life and survival: a large population-based cohort study. Haematologica 104, 468–476. 30309850 10.3324/haematol.2018.195552 PMC6395328

[6] Safiri S , Kolahi AA , Noori M et al. (2021) Burden of anemia and its underlying causes in 204 countries and territories, 1990–2019: results from the global burden of disease study 2019. J Hematol Oncol 14, 185. 34736513 10.1186/s13045-021-01202-2 PMC8567696

[7] Li M , Hu Y , Mao D et al. (2017) Prevalence of anemia among Chinese rural residents. Nutrients 9, 192. 28245576 10.3390/nu9030192 PMC5372855

[8] WHO (2011) Haemoglobin Concentrations for the Diagnosis of Anaemia and Assessment of Severity. Vitamin and Mineral Nutrition Information System. Geneva: World Health Organization.

[9] Pasricha SR , Colman K , Centeno-Tablante E et al. (2018) Revisiting WHO haemoglobin thresholds to define anaemia in clinical medicine and public health. Lancet Haematol 5, e60–e62. 29406148 10.1016/S2352-3026(18)30004-8

[10] Addo OY , Yu EX , Williams AM et al. (2021) Evaluation of hemoglobin cutoff levels to define anemia among healthy individuals. JAMA Netw Open 4, e2119123. 34357395 10.1001/jamanetworkopen.2021.19123 PMC8346941

[11] Didzun O , De Neve JW , Awasthi A et al. (2019) Anaemia among men in India: a nationally representative cross-sectional study. Lancet Glob Health 7, e1685–e1694. 31708149 10.1016/S2214-109X(19)30440-1

[12] Tong TYN , Key TJ , Gaitskell K et al. (2019) Hematological parameters and prevalence of anemia in white and British Indian vegetarians and nonvegetarians in the UK Biobank. Am J Clin Nutr 110, 461–472. 31190054 10.1093/ajcn/nqz072 PMC6669054

[13] Cascio MJ & DeLoughery TG (2017) Anemia: evaluation and diagnostic tests. Med Clin North Am 101, 263–284. 28189170 10.1016/j.mcna.2016.09.003

[14] Tefferi A (2003) Anemia in adults: a contemporary approach to diagnosis. Mayo Clin Proc 78, 1274–1280. 14531486 10.4065/78.10.1274

[15] Liu X , Mao Z , Li Y et al. (2019) Cohort profile: the Henan rural cohort: a prospective study of chronic non-communicable diseases. Int J Epidemiol 48, 1756. 30915440 10.1093/ije/dyz039

[16] WHO (1997) Guidelines for Controlling and Monitoring the Tobacco Epidemic. Geneva: Tobacco or Health Programme, WHO.

[17] WHO (2000) International Guide for Monitoring Alcohol Consumption and Related Harm. Geneva: World Health Organization.

[18] Chinese Nutrition Society (2011) The Dietary Guidelines for Chinese Residents. Lhasa: The Tibet People’s Publishing House.

[19] Craig CL , Marshall AL , Sjöström M et al. (2003) International physical activity questionnaire: 12-country reliability and validity. Med Sci Sports Exerc 35, 1381–1395. 12900694 10.1249/01.MSS.0000078924.61453.FB

[20] Mi YJ , Zhang B , Wang HJ et al. (2015) Prevalence and secular trends in obesity among Chinese adults, 1991–2011. Am J Prev Med 49, 661–669. 26275960 10.1016/j.amepre.2015.05.005 PMC4615397

[21] Chobanian AV , Bakris GL , Black HR et al. (2003) Seventh report of the joint national committee on prevention, detection, evaluation, and treatment of high blood pressure. Hypertension 42, 1206–1252. 14656957 10.1161/01.HYP.0000107251.49515.c2

[22] Working Group on Obesity in China (2004) The guidelines for prevention and control of overweight and obesity in Chinese adults. Acta Nutr Sin 26, 1–4.

[23] American Diabetes Association (2009) Diagnosis and classification of diabetes mellitus. Diabetes Care 32, Suppl. 1, S62–S67. 19118289 10.2337/dc09-S062 PMC2613584

[24] Wang Y , Li Y , Liu X et al. (2019) Prevalence and influencing factors of coronary heart disease and stroke in Chinese rural adults: the Henan rural cohort study. Front Public Health 7, 411. 32039127 10.3389/fpubh.2019.00411 PMC6985463

[25] Levey AS , Stevens LA , Schmid CH et al. (2009) A new equation to estimate glomerular filtration rate. Ann Intern Med 150, 604–612. 19414839 10.7326/0003-4819-150-9-200905050-00006 PMC2763564

[26] Duan J , Wang C , Liu D et al. (2019) Prevalence and risk factors of chronic kidney disease and diabetic kidney disease in Chinese rural residents: a cross-sectional survey. Sci Rep 9, 10408. 31320683 10.1038/s41598-019-46857-7 PMC6639314

[27] Martinsson A , Andersson C , Andell P et al. (2014) Anemia in the general population: prevalence, clinical correlates and prognostic impact. Eur J Epidemiol 29, 489–498. 24952166 10.1007/s10654-014-9929-9

[28] Bessman JD , Gilmer PR Jr & Gardner FH (1983) Improved classification of anemias by MCV and RDW. Am J Clin Pathol 80, 322–326. 6881096 10.1093/ajcp/80.3.322

[29] Xia W , Chen M , Wang X et al. (2015) Clinical Hematological Examination Techniques. Beijing: People’s Medical Publishing House.

[30] Abdullah N , Ismail N , Abd Jalal N et al. (2020) Prevalence of anaemia and associated risk factors amongst the Malaysian cohort participants. Ann Hematol 99, 2521–2527. 32975589 10.1007/s00277-020-04279-w

[31] Zakai NA , McClure LA , Prineas A et al. (2009) Correlates of anemia in American blacks and whites: the REGARDS renal ancillary study. Am J Epidemiol 169, 355–364. 19066309 10.1093/aje/kwn355 PMC2720717

[32] Bonomini M , Del Vecchio L , Sirolli V et al. (2016) New treatment approaches for the anemia of CKD. Am J Kidney Dis 67, 133–142. 26372086 10.1053/j.ajkd.2015.06.030

[33] Nah EH , Cho S , Kim S et al. (2020) Distribution of hemoglobin levels and prevalence of anemia according to sex, age group, and region in 13 Korean cities. Int J Lab Hematol 42, 223–229. 32048801 10.1111/ijlh.13160

[34] Balarajan Y , Ramakrishnan U , Ozaltin E et al. (2011) Anaemia in low-income and middle-income countries. Lancet 378, 2123–2135. 21813172 10.1016/S0140-6736(10)62304-5

[35] Daru J , Zamora J , Fernández-Félix BM et al. (2018) Risk of maternal mortality in women with severe anaemia during pregnancy and post partum: a multilevel analysis. Lancet Glob Health 6, e548–e554. 29571592 10.1016/S2214-109X(18)30078-0

[36] Culleton BF , Manns BJ , Zhang J et al. (2006) Impact of anemia on hospitalization and mortality in older adults. Blood 107, 3841–3846. 16403909 10.1182/blood-2005-10-4308

[37] Chueh HW , Jung HL , Shim YJ et al. (2020) High anemia prevalence in Korean older adults, an advent healthcare problem: 2007–2016 KNHANES. BMC Geriatr 20, 509. 33243179 10.1186/s12877-020-01918-9 PMC7689998

[38] Lee CT , Chen MZ , Yip CYC et al. (2021) Prevalence of anemia and its association with frailty, physical function and cognition in community-dwelling older adults: findings from the HOPE study. J Nutr Health Aging 25, 679–687.3394963710.1007/s12603-021-1625-3

[39] Lee YG , Chang Y , Kang J et al. (2019) Risk factors for incident anemia of chronic diseases: a cohort study. PLoS ONE 14, e0216062. 31059543 10.1371/journal.pone.0216062 PMC6502324

[40] Shimizu Y , Sato S , Koyamatsu J et al. (2014) Associations between renal impairment and anemia in older, rural Japanese men: the Nagasaki Island study. J Physiol Anthropol 33, 7. 24742197 10.1186/1880-6805-33-7 PMC4012527

[41] Yoon H , Lee JH , Kim GS et al. (2018) The relationship between anemia and pulse pressure and hypertension: the Korea national health and nutrition examination survey 2010–2012. Clin Exp Hypertens 40, 650–655. 29319360 10.1080/10641963.2017.1416123

[42] Mirza AS , Chen L , Amirzadeh S et al. (2019) Health disparities and chronic disease associated with anemia in free clinics: a retrospective study of uninsured patients in Tampa Bay. Postgrad Med 131, 612–618. 31524033 10.1080/00325481.2019.1668241

[43] Lakew Y , Biadgilign S & Haile D (2015) Anaemia prevalence and associated factors among lactating mothers in Ethiopia: evidence from the 2005 and 2011 demographic and health surveys. BMJ Open 5, e006001. 10.1136/bmjopen-2014-006001 PMC4401847 25872935

[44] Keohane C , McMullin MF & Harrison C (2013) The diagnosis and management of erythrocytosis. BMJ 347, f6667. 24246666 10.1136/bmj.f6667

[45] Gordeuk VR , Key NS & Prchal JT (2019) Re-evaluation of hematocrit as a determinant of thrombotic risk in erythrocytosis. Haematologica 104, 653–658. 30872370 10.3324/haematol.2018.210732 PMC6442963

[46] Wouters H , Mulder R , van Zeventer IA et al. (2020) Erythrocytosis in the general population: clinical characteristics and association with clonal hematopoiesis. Blood Adv 4, 6353–6363. 33351130 10.1182/bloodadvances.2020003323 PMC7757002

[47] Oshima M , Shimizu M , Yamanouchi M et al. (2021) Trajectories of kidney function in diabetes: a clinicopathological update. Nat Rev Nephrol 17, 740–750. 34363037 10.1038/s41581-021-00462-y

[48] Ganz T (2019) Anemia of inflammation. N Engl J Med 381, 1148–1157. 31532961 10.1056/NEJMra1804281

[49] Koury MJ & Haase VH (2015) Anaemia in kidney disease: harnessing hypoxia responses for therapy. Nat Rev Nephrol 11, 394–410. 26055355 10.1038/nrneph.2015.82 PMC4497972

[50] Shih HM , Wu CJ & Lin SL (2018) Physiology and pathophysiology of renal erythropoietin-producing cells. J Formos Med Assoc 117, 955–963. 29655605 10.1016/j.jfma.2018.03.017

