# Supplementary material for: Prevalence and morphological subtype distributions of anaemia in a Chinese rural population: the Henan Rural Cohort study
Source: Public Health Nutr. 2023 Feb 15;26(6):1254–63. doi: 10.1017/S1368980023000319 (PMC10346018; doi:10.1017/S1368980023000319)
Supplement: Supplementary file 1 [file S1368980023000319sup.zip › S1368980023000319sup001.docx]

Supplementary Table 1 The classified methods proposed by Bessman.

|  | MCV (fL) | RDW-CV (%) |
| --- | --- | --- |
| Microcytic homogeneous anemia | <80 | 11.5~14.5 |
| Microcytic heterogeneous anemia | <80 | >14.5 |
| Normocytic homogeneous anemia | 80~100 | 11.5~14.5 |
| Normocytic heterogeneous anemia | 80~100 | >14.5 |
| Macrocytic homogeneous anemia | >100 | 11.5~14.5 |
| Macrocytic heterogeneous anemia | >100 | >14.5 |

Abbreviation: MCV, mean corpuscular volume; RDW-CV, red blood cell volume distribution width coefficient of variation.

Supplementary Table 2 The classified methods proposed by Wintrobe.

|  | MCV (fL) | MCH (pg) | MCHC (g/L) |
| --- | --- | --- | --- |
| Normocytic normochromic anemia | 80~100 | 27~34 | 320~360 |
| Macrocytic normochromic anemia | >100 | >34 | 320~360 |
| Simple microcytic anemia | <80 | <27 | 320~360 |
| Microcytic hypochromic anemia | <80 | <27 | <320 |

Abbreviation: MCV, mean corpuscular volume; MCH, mean corpuscular hemoglobin; MCHC, mean corpuscular hemoglobin concentration.

Supplementary Table 3 Characteristics of study participants according to the Chinese definition.

| Variables | Men (N=13574) | | |  | Women (N=20011) | | |
| --- | --- | --- | --- | --- | --- | --- | --- |
|  | Anemia | Non-anemia | *P*-value |  | Anemia | Non-anemia | *P*-value |
| Age (years), mean ± SD | 64.23±9.22 | 56.11±12.38 | <0.001 |  | 48.36±10.82 | 55.06±12.15 | <0.001 |
| Education level, n (%) |  |  | <0.001 |  |  |  | <0.001 |
| Primary school or below | 120 (54.55) | 4357 (32.63) |  |  | 536 (41.88) | 9669 (51.62) |  |
| Junior school | 79 (35.91) | 6176 (46.25) |  |  | 561 (43.83) | 6605 (35.26) |  |
| High school or above | 21 (9.55) | 2821 (21.12) |  |  | 183 (14.30) | 2457 (13.12) |  |
| Marital status, n (%) |  |  | <0.001 |  |  |  | <0.001 |
| Married/Cohabiting | 171 (77.73) | 12095 (90.57) |  |  | 1204 (94.06) | 16817 (89.78) |  |
| Widowed/Single/Divorced/Separated | 49 (22.27) | 1259 (9.43) |  |  | 76 (5.94) | 1914 (10.22) |  |
| Per capita monthly income, n (%) |  |  | 0.001 |  |  |  | 0.189 |
| <500 RMB | 99 (45.00) | 4775 (35.76) |  |  | 419 (32.73) | 6590 (35.18) |  |
| 500-1000 RMB | 74 (33.64) | 4201 (31.46) |  |  | 435 (33.98) | 6222 (33.22) |  |
| ≥1000 RMB | 47 (21.36) | 4378 (32.78) |  |  | 426 (33.28) | 5919 (31.6) |  |
| Smoking status, n (%) |  |  | 0.002 |  |  |  | 0.320 |
| Never | 70 (31.82) | 4231 (31.68) |  |  | 1279 (99.92) | 18655 (99.59) |  |
| Light | 13 (5.91) | 1910 (14.30) |  |  | 1 (0.08) | 44 (0.23) |  |
| Moderate | 23 (10.45) | 1534 (11.49) |  |  | 0 (0) | 12 (0.06) |  |
| Heavy | 114 (51.82) | 5679 (42.53) |  |  | 0 (0) | 20 (0.11) |  |
| Drinking status, n (%) |  |  | 0.079 |  |  |  | 0.847 |
| Never | 124 (56.36) | 6350 (47.55) |  |  | 1250 (97.66) | 18284 (97.61) |  |
| Light | 58 (26.36) | 4183 (31.32) |  |  | 24 (1.88) | 355 (1.90) |  |
| Moderate | 20 (9.09) | 1538 (11.52) |  |  | 5 (0.39) | 60 (0.32) |  |
| Heavy | 18 (8.18) | 1283 (9.61) |  |  | 1 (0.08) | 32 (0.17) |  |
| Physical activity, n (%) |  |  | 0.005 |  |  |  | 0.619 |
| Low | 86 (39.09) | 4659 (34.89) |  |  | 372 (29.06) | 5604 (29.92) |  |
| Moderate | 76 (34.55) | 3763 (28.18) |  |  | 582 (45.47) | 8255 (44.07) |  |
| High | 58 (26.36) | 4932 (36.93) |  |  | 326 (25.47) | 4872 (26.01) |  |
| High fat diet, n (%) |  |  | 0.012 |  |  |  | 0.002 |
| No | 182 (82.73) | 10068 (75.39) |  |  | 1053 (82.27) | 15996 (85.4) |  |
| Yes | 38 (17.27) | 3286 (24.61) |  |  | 227 (17.73) | 2735 (14.6) |  |
| Adequate vegetable and fruit intake, n (%) |  |  | 0.481 |  |  |  | 0.213 |
| No | 115 (52.27) | 7298 (54.66) |  |  | 688 (53.75) | 10403 (55.54) |  |
| Yes | 105 (47.73) | 6054 (45.34) |  |  | 592 (46.25) | 8328 (44.46) |  |
| Hypertension, n (%) | 71 (32.27) | 4333 (32.45) | 0.956 |  | 211 (16.48) | 6164 (32.91) | <0.001 |
| T2DM, n (%) | 26 (11.82) | 1156 (8.66) | 0.099 |  | 56 (4.38) | 1782 (9.51) | <0.001 |
| Dyslipidemia, n (%) | 80 (36.36) | 5442 (40.75) | 0.189 |  | 339 (26.48) | 6976 (37.24) | <0.001 |
| BMI (kg/m^2^), mean ± SD | 22.87±3.77 | 24.57±3.46 | <0.001 |  | 24.59±3.55 | 24.98±3.61 | <0.001 |
| eGFR (mL/min/1.73 m^2^), mean ± SD | 91.04±17.23 | 99.01±12.99 | <0.001 |  | 106.18±14.81 | 100.37±13.48 | <0.001 |
| Hemoglobin (g/L), mean ± SD | 109.15±12.83 | 149.08±12.85 | <0.001 |  | 96.29±11.71 | 132.58±12.49 | <0.001 |

Abbreviation: WHO, World Health Organization; SD, standard deviation; RMB, renminbi, the Chinese currency, and the average exchange rate for USD/RMB from 2015 to 2017 is 6.54; T2DM, type 2 diabetes mellitus; BMI, body mass index; eGFR, estimated glomerular filtration rate.

Supplementary Table 4 The prevalence and 95% confidence intervals for anemia among characteristics according to the Chinese definition.

| Variables | Men (N=13574) | | |  | Women (N=20011) | | |
| --- | --- | --- | --- | --- | --- | --- | --- |
|  | N | Prevalence (95% *CI*) | *P*-value |  | N | Prevalence (95% *CI*) | *P*-value |
| Education level |  |  | <0.001 |  |  |  | <0.001 |
| Primary school or below | 4477 | 2.68 (2.21-3.15) |  |  | 10205 | 5.25 (4.82-5.69) |  |
| Junior school | 6255 | 1.26 (0.99-1.54) |  |  | 7166 | 7.83 (7.21-8.45) |  |
| High school or above | 2842 | 0.74 (0.42-1.05) |  |  | 2640 | 6.93 (5.96-7.90) |  |
| Marital status |  |  | <0.001 |  |  |  | <0.001 |
| Married/Cohabiting | 12266 | 1.39 (1.19-1.60) |  |  | 18021 | 6.68 (6.32-7.05) |  |
| Widowed/Single/Divorced/Separated | 1308 | 3.75 (2.72-4.78) |  |  | 1990 | 3.82 (2.98-4.66) |  |
| Per capita monthly income |  |  | 0.001 |  |  |  | 0.189 |
| <500 RMB | 4874 | 2.03 (1.64-2.43) |  |  | 7009 | 5.98 (5.42-6.53) |  |
| 500-1000 RMB | 4275 | 1.73 (1.34-2.12) |  |  | 6657 | 6.53 (5.94-7.13) |  |
| ≥1000 RMB | 4425 | 1.06 (0.76-1.36) |  |  | 6345 | 6.71 (6.10-7.33) |  |
| Smoking status |  |  | 0.002 |  |  |  | 0.320 |
| Never | 4301 | 1.63 (1.25-2.01) |  |  | 19934 | 6.42 (6.08-6.76) |  |
| Light | 1923 | 0.68 (0.31-1.04) |  |  | 45 | 2.22 (-2.26-6.7) |  |
| Moderate | 1557 | 1.48 (0.88-2.08) |  |  | 12 | 0 (0-0) |  |
| Heavy | 5793 | 1.97 (1.61-2.33) |  |  | 20 | 0 (0-0) |  |
| Drinking status |  |  | 0.079 |  |  |  | 0.847 |
| Never | 6474 | 1.92 (1.58-2.25) |  |  | 19534 | 6.4 (6.06-6.74) |  |
| Light | 4241 | 1.37 (1.02-1.72) |  |  | 379 | 6.33 (3.87-8.80) |  |
| Moderate | 1558 | 1.28 (0.72-1.84) |  |  | 65 | 7.69 (1.04-14.35) |  |
| Heavy | 1301 | 1.38 (0.75-2.02) |  |  | 33 | 3.03 (-3.14-9.20) |  |
| Physical activity |  |  | 0.005 |  |  |  | 0.619 |
| Low | 4745 | 1.81 (1.43-2.19) |  |  | 5976 | 6.22 (5.61-6.84) |  |
| Moderate | 3839 | 1.98 (1.54-2.42) |  |  | 8837 | 6.59 (6.07-7.10) |  |
| High | 4990 | 1.16 (0.86-1.46) |  |  | 5198 | 6.27 (5.61-6.93) |  |
| High fat diet |  |  | 0.012 |  |  |  | 0.002 |
| No | 10250 | 1.78 (1.52-2.03) |  |  | 17049 | 6.18 (5.81-6.54) |  |
| Yes | 3324 | 1.14 (0.78-1.50) |  |  | 2962 | 7.66 (6.71-8.62) |  |
| Adequate vegetable and fruit intake |  |  | 0.481 |  |  |  | 0.213 |
| No | 7413 | 1.55 (1.27-1.83) |  |  | 11091 | 6.2 (5.75-6.65) |  |
| Yes | 6159 | 1.7 (1.38-2.03) |  |  | 8920 | 6.64 (6.12-7.15) |  |
| BMI |  |  | <0.001 |  |  |  | 0.001 |
| Underweight | 370 | 5.68 (3.31-8.04) |  |  | 463 | 7.99 (5.51-10.47) |  |
| Normal | 5851 | 2.1 (1.73-2.47) |  |  | 7810 | 7.14 (6.57-7.72) |  |
| Overweight | 5171 | 0.85 (0.60-1.10) |  |  | 7999 | 6.03 (5.50-6.55) |  |
| Obesity | 2141 | 1.31 (0.83-1.79) |  |  | 3687 | 5.4 (4.67-6.13) |  |
| Hypertension | 4404 | 1.61 (1.24-1.98) | 0.956 |  | 6375 | 7.84 (7.39-8.29) | <0.001 |
| T2DM | 1182 | 2.2 (1.36-3.04) | 0.099 |  | 1838 | 3.05 (2.26-3.83) | <0.001 |
| Dyslipidemia | 5522 | 1.45 (1.13-1.76) | 0.189 |  | 7315 | 4.63 (4.15-5.12) | <0.001 |
| Renal damage | 120 | 11.67 (5.84-17.49) | <0.001 |  | 160 | 13.13 (7.84-18.41) | <0.001 |

Abbreviation: CI, confidence interval; RMB, renminbi, the Chinese currency, and the average exchange rate for USD/RMB from 2015 to 2017 is 6.54; T2DM, type 2 diabetes mellitus; BMI, body mass index.

Supplementary Table 5 Gender-specific multivariable logistic regression analysis for the influencing factors for anemia according to the Chinese definition.

| Variables | Men | |  | Women | |
| --- | --- | --- | --- | --- | --- |
|  | a*OR* (95% *CI*) | *P*-value |  | a*OR* (95% *CI*) | *P*-value |
| Age | 1.047 (1.03, 1.064) | <0.001 |  | 0.957 (0.952, 0.963) | <0.001 |
| Education level |  |  |  |  |  |
| primary school or below | Ref (1.000) |  |  | Ref (1.000) |  |
| junior school | 0.730 (0.535, 0.997) | 0.048 |  | 0.906 (0.790, 1.039) | 0.156 |
| high school or above | 0.589 (0.357, 0.970) | 0.038 |  | 0.569 (0.463, 0.699) | <0.001 |
| Marital status |  |  |  |  |  |
| married/cohabiting | Ref (1.000) |  |  | Ref (1.000) |  |
| widowed/single/divorced/separated | 1.760 (1.244, 2.490) | 0.001 |  | 0.752 (0.589, 0.961) | 0.022 |
| Per capita monthly income |  |  |  |  |  |
| <500 RMB | Ref (1.000) |  |  | Ref (1.000) |  |
| 500-1000 RMB | 1.165 (0.849, 1.599) | 0.344 |  | 0.907 (0.787, 1.046) | 0.180 |
| ≥1000 RMB | 0.910 (0.629, 1.315) | 0.614 |  | 0.862 (0.744, 0.997) | 0.046 |
| Smoking status |  |  |  |  |  |
| Never | Ref (1.000) |  |  | Ref (1.000) |  |
| Light | 0.445 (0.238, 0.833) | 0.011 |  | 0.403 (0.054, 2.99) | 0.374 |
| Moderate | 0.977 (0.594, 1.608) | 0.927 |  | 0 (0, 0) | 0.999 |
| Heavy | 1.054 (0.763, 1.456) | 0.751 |  | 0 (0, 0) | 0.998 |
| Drinking status |  |  |  |  |  |
| Never | Ref (1.000) |  |  | Ref (1.000) |  |
| Light | 1.086 (0.777, 1.517) | 0.631 |  | 0.781 (0.512, 1.193) | 0.253 |
| Moderate | 1.049 (0.638, 1.725) | 0.850 |  | 1.230 (0.486, 3.114) | 0.662 |
| Heavy | 1.002 (0.594, 1.690) | 0.993 |  | 0.504 (0.067, 3.779) | 0.505 |
| High fat diet | 0.880 (0.604, 1.282) | 0.506 |  | 1.030 (0.882, 1.203) | 0.709 |
| Adequate vegetable and fruit intake | 1.138 (0.861, 1.504) | 0.362 |  | 0.975 (0.866, 1.097) | 0.670 |
| Physical activity |  |  |  |  |  |
| Low | Ref (1.000) |  |  | Ref (1.000) |  |
| Moderate | 1.178 (0.850, 1.631) | 0.325 |  | 0.958 (0.834, 1.101) | 0.548 |
| High | 0.765 (0.538, 1.086) | 0.134 |  | 0.974 (0.831, 1.141) | 0.742 |
| BMI group |  |  |  |  |  |
| Normal | Ref (1.000) |  |  | Ref (1.000) |  |
| Underweight | 2.175 (1.330, 3.555) | 0.002 |  | 1.073 (0.752, 1.529) | 0.699 |
| Overweight | 0.449 (0.312, 0.645) | <0.001 |  | 0.962 (0.845, 1.096) | 0.559 |
| Obesity | 0.763 (0.483, 1.206) | 0.248 |  | 0.946 (0.794, 1.127) | 0.534 |
| Hypertension | 0.834 (0.610, 1.142) | 0.258 |  | 0.605 (0.513, 0.713) | <0.001 |
| T2DM | 1.592 (1.033, 2.453) | 0.035 |  | 0.684 (0.517, 0.905) | 0.008 |
| Dyslipidemia | 1.144 (0.843, 1.552) | 0.389 |  | 0.800 (0.699, 0.916) | 0.001 |
| Renal damage | 5.036 (2.702, 9.386) | <0.001 |  | 5.557 (3.438, 8.983) | <0.001 |

Abbreviation: WHO, World Health Organization; OR, odds ratio; CI, confidence interval; RMB, renminbi, the Chinese currency, and the average exchange rate for USD/RMB from 2015 to 2017 is 6.54; BMI, body mass index; T2DM, type 2 diabetes mellitus.

Supplementary Table 6 Multivariable logistic regression analysis for the influencing factors for anemia stratified by menopause status according to the WHO definition.

| Variables | Pre-menopause Women | |  | Post-menopause Women | |
| --- | --- | --- | --- | --- | --- |
|  | a*OR* (95% *CI*) | *P*-value |  | a*OR* (95% *CI*) | *P*-value |
| Age | 1.017 (1.009, 1.025) | <0.001 |  | 1.016 (1.007, 1.025) | <0.001 |
| Education level |  |  |  |  |  |
| primary school or below | Ref (1.000) |  |  | Ref (1.000) |  |
| junior school | 0.906 (0.799, 1.027) | 0.121 |  | 0.792 (0.684, 0.917) | 0.002 |
| high school or above | 0.771 (0.648, 0.917) | 0.003 |  | 0.682 (0.531, 0.876) | 0.003 |
| Marital status |  |  |  |  |  |
| married/cohabiting | Ref (1.000) |  |  | Ref (1.000) |  |
| widowed/single/divorced/separated | 0.983 (0.722, 1.338) | 0.913 |  | 1.082 (0.915, 1.279) | 0.357 |
| Per capita monthly income |  |  |  |  |  |
| <500 RMB | Ref (1.000) |  |  | Ref (1.000) |  |
| 500-1000 RMB | 0.938 (0.818, 1.076) | 0.361 |  | 0.831 (0.722, 0.956) | 0.010 |
| ≥1000 RMB | 0.925 (0.806, 1.061) | 0.264 |  | 1.008 (0.873, 1.162) | 0.918 |
| Smoking status |  |  |  |  |  |
| Never | Ref (1.000) |  |  | Ref (1.000) |  |
| Light | 1.174 (0.303, 4.542) | 0.817 |  | 0.857 (0.246, 2.988) | 0.808 |
| Moderate | 0 (0, 0) | 1.000 |  | 0.528 (0.065, 4.326) | 0.552 |
| Heavy | 0 (0, 0) | 1.000 |  | 1.528 (0.413, 5.658) | 0.525 |
| Drinking status |  |  |  |  |  |
| Never | Ref (1.000) |  |  | Ref (1.000) |  |
| Light | 0.638 (0.449, 0.907) | 0.012 |  | 1.189 (0.750, 1.887) | 0.462 |
| Moderate | 0.349 (0.103, 1.178) | 0.090 |  | 0.783 (0.269, 2.279) | 0.653 |
| Heavy | 0.432 (0.095, 1.956) | 0.276 |  | 0.576 (0.073, 4.547) | 0.601 |
| High fat diet | 0.955 (0.835, 1.093) | 0.504 |  | 0.995 (0.828, 1.196) | 0.959 |
| Adequate vegetable and fruit intake | 1.077 (0.967, 1.200) | 0.179 |  | 1.240 (1.101, 1.396) | <0.001 |
| Physical activity |  |  |  |  |  |
| Low | Ref (1.000) |  |  | Ref (1.000) |  |
| Moderate | 1.060 (0.932, 1.206) | 0.372 |  | 1.007 (0.875, 1.159) | 0.922 |
| High | 1.057 (0.910, 1.227) | 0.469 |  | 0.964 (0.823, 1.129) | 0.650 |
| BMI group |  |  |  |  |  |
| Normal | Ref (1.000) |  |  | Ref (1.000) |  |
| Underweight | 1.321 (0.917, 1.903) | 0.135 |  | 1.637 (1.237, 2.166) | 0.001 |
| Overweight | 0.910 (0.807, 1.026) | 0.123 |  | 0.616 (0.540, 0.701) | <0.001 |
| Obesity | 0.722 (0.612, 0.852) | <0.001 |  | 0.482 (0.399, 0.582) | <0.001 |
| Hypertension | 0.681 (0.573, 0.809) | <0.001 |  | 0.651 (0.571, 0.741) | <0.001 |
| T2DM | 0.743 (0.544, 1.015) | 0.062 |  | 0.833 (0.683, 1.017) | 0.073 |
| Dyslipidemia | 0.925 (0.814, 1.052) | 0.237 |  | 0.704 (0.621, 0.798) | <0.001 |
| Renal damage | 1.699 (0.153, 18.917) | 0.666 |  | 5.233 (3.689, 7.424) | <0.001 |

Abbreviation: WHO, World Health Organization; OR, odds ratio; CI, confidence interval; RMB, renminbi, the Chinese currency, and the average exchange rate for USD/RMB from 2015 to 2017 is 6.54; BMI, body mass index; T2DM, type 2 diabetes mellitus.

Supplementary Table 7 Multivariable logistic regression analysis for the influencing factors for anemia stratified by menopause status according to the Chinese definition.

| Variables | Pre-menopause Women | |  | Post-menopause Women | |
| --- | --- | --- | --- | --- | --- |
|  | a*OR* (95% *CI*) | *P*-value |  | a*OR* (95% *CI*) | *P*-value |
| Age | 1.028 (1.018, 1.038) | <0.001 |  | 1.006 (0.989, 1.024) | 0.482 |
| Education level |  |  |  |  |  |
| primary school or below | Ref (1.000) |  |  | Ref (1.000) |  |
| junior school | 0.870 (0.743, 1.018) | 0.083 |  | 0.846 (0.630, 1.136) | 0.267 |
| high school or above | 0.762 (0.608, 0.955) | 0.018 |  | 0.831 (0.512, 1.348) | 0.453 |
| Marital status |  |  |  |  |  |
| married/cohabiting | Ref (1.000) |  |  | Ref (1.000) |  |
| widowed/single/divorced/separated | 0.731 (0.466, 1.147) | 0.173 |  | 1.172 (0.847, 1.621) | 0.339 |
| Per capita monthly income |  |  |  |  |  |
| <500 RMB | Ref (1.000) |  |  | Ref (1.000) |  |
| 500-1000 RMB | 0.922 (0.775, 1.096) | 0.356 |  | 0.797 (0.604, 1.052) | 0.109 |
| ≥1000 RMB | 0.877 (0.735, 1.045) | 0.143 |  | 0.829 (0.619, 1.110) | 0.209 |
| Smoking status |  |  |  |  |  |
| Never | Ref (1.000) |  |  | Ref (1.000) |  |
| Light | 0.600 (0.075, 4.774) | 0.629 |  | 0 (0, 0) | 0.998 |
| Moderate | 0 (0, 0) | 1.000 |  | 0 (0, 0) | 0.999 |
| Heavy | 0 (0, 0) | 1.000 |  | 0 (0, 0) | 0.998 |
| Drinking status |  |  |  |  |  |
| Never | Ref (1.000) |  |  | Ref (1.000) |  |
| Light | 0.635 (0.393, 1.026) | 0.064 |  | 1.262 (0.511, 3.112) | 0.614 |
| Moderate | 0.834 (0.245, 2.840) | 0.771 |  | 1.133 (0.153, 8.365) | 0.903 |
| Heavy | 0.490 (0.063, 3.789) | 0.494 |  | 0 (0, 0) | 0.998 |
| High fat diet | 1.045 (0.879, 1.244) | 0.616 |  | 0.858 (0.581, 1.267) | 0.442 |
| Adequate vegetable and fruit intake | 0.884 (0.768, 1.016) | 0.083 |  | 1.216 (0.959, 1.542) | 0.107 |
| Physical activity |  |  |  |  |  |
| Low | Ref (1.000) |  |  | Ref (1.000) |  |
| Moderate | 1.011 (0.856, 1.193) | 0.899 |  | 0.911 (0.689, 1.204) | 0.512 |
| High | 0.990 (0.818, 1.198) | 0.919 |  | 0.912 (0.667, 1.247) | 0.564 |
| BMI group |  |  |  |  |  |
| Normal | Ref (1.000) |  |  | Ref (1.000) |  |
| Underweight | 1.171 (0.709, 1.933) | 0.538 |  | 1.553 (0.918, 2.626) | 0.100 |
| Overweight | 1.000 (0.857, 1.167) | 0.999 |  | 0.638 (0.488, 0.835) | 0.001 |
| Obesity | 0.895 (0.726, 1.103) | 0.297 |  | 0.687 (0.483, 0.978) | 0.037 |
| Hypertension | 0.699 (0.560, 0.871) | 0.001 |  | 0.661 (0.509, 0.859) | 0.002 |
| T2DM | 0.537 (0.343, 0.841) | 0.007 |  | 1.101 (0.759, 1.597) | 0.611 |
| Dyslipidemia | 0.985 (0.837, 1.158) | 0.852 |  | 0.575 (0.442, 0.748) | <0.001 |
| Renal damage | 3.905 (0.346, 44.097) | 0.271 |  | 6.719 (4.039, 11.177) | <0.001 |

Abbreviation: WHO, World Health Organization; OR, odds ratio; CI, confidence interval; RMB, renminbi, the Chinese currency, and the average exchange rate for USD/RMB from 2015 to 2017 is 6.54; BMI, body mass index; T2DM, type 2 diabetes mellitus.

**Supplementary Figure legends**

**Supplementary Figure 1 The age-standardized prevalence of morphological subtypes of anemia according to the Chinese definition.** (The age-standardized prevalence of morphological subtypes of anemia classified based on mean corpuscular volume were showed in Panel A. Panels B and C showed the age-standardized prevalence of morphological subtypes of anemia according to the methods proposed by Bessman and Wintrobe, respectively.)

**Supplementary Figure 2 Age-specific prevalence of morphological subtypes of anemia by the WHO definition among Chinese rural population.** (The age-standardized prevalence of morphological subtypes of anemia classified based on mean corpuscular volume were showed in Panel A. Panels B and C showed the age-standardized prevalence of morphological subtypes of anemia according to the methods proposed by Bessman and Wintrobe, respectively.)

**Supplementary Figure 3** **Age-specific prevalence of morphological subtypes of anemia by the Chinese definition among Chinese rural population.** (The age-standardized prevalence of morphological subtypes of anemia classified based on mean corpuscular volume were showed in Panel A. Panels B and C showed the age-standardized prevalence of morphological subtypes of anemia according to the methods proposed by Bessman and Wintrobe, respectively.)
