# Supplementary figures and images for: Prevalence and morphological subtype distributions of anaemia in a Chinese rural population: the Henan Rural Cohort study
Source: Public Health Nutr. 2023 Feb 15;26(6):1254–63. doi: 10.1017/S1368980023000319 (PMC10346018; doi:10.1017/S1368980023000319)

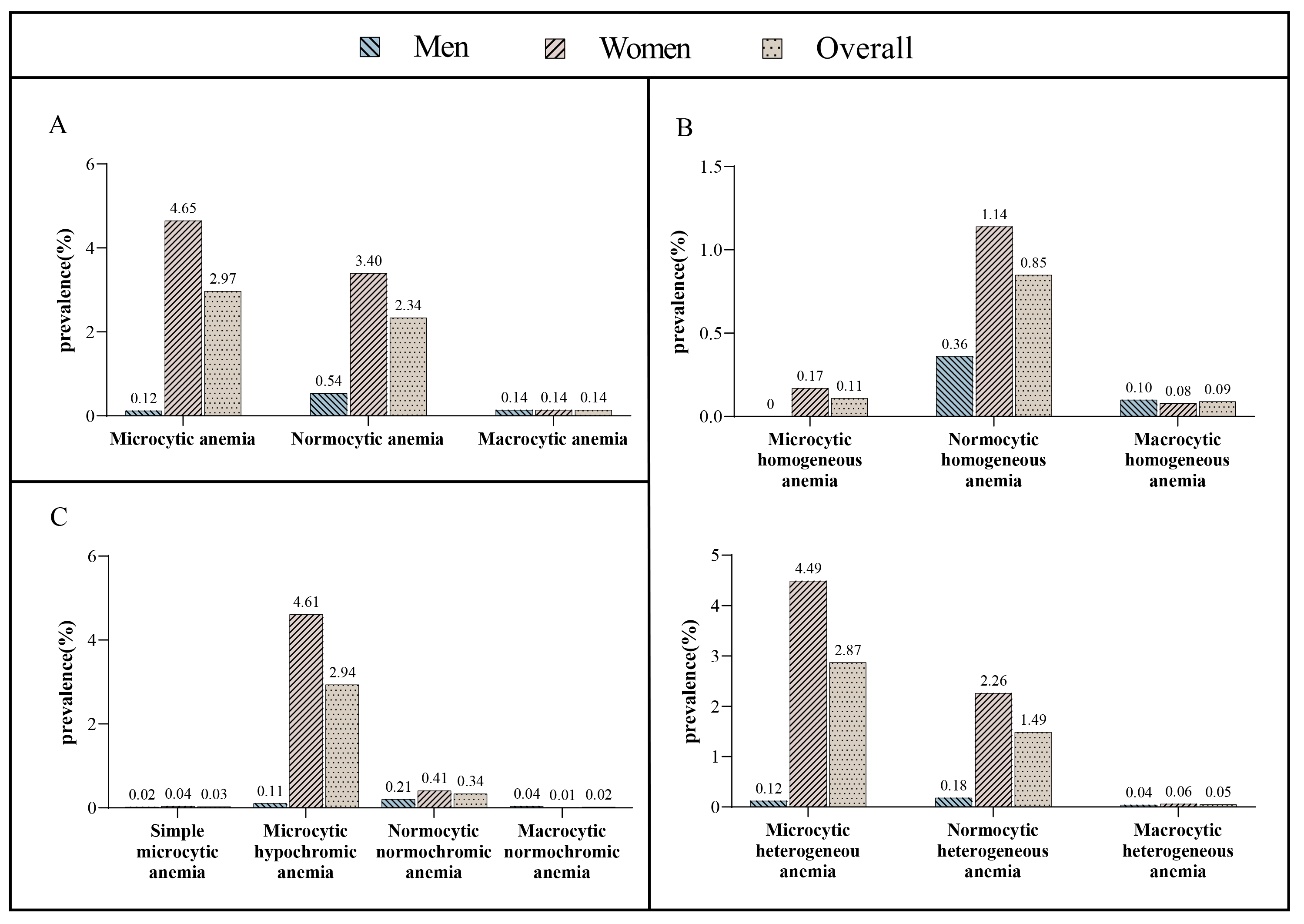

Supplement: Supplementary file 1 [file S1368980023000319sup.zip › S1368980023000319sup003.tif]
